# The effect of platelet enriched plasma on clinical outcomes in patients with femoroacetabular impingement following arthroscopic labral repair and femoral neck osteoplasty

**DOI:** 10.1093/jhps/hnv023

**Published:** 2015-03-30

**Authors:** Russell LaFrance, Raymond Kenney, Brian Giordano, Karen Mohr, Jennifer Cabrera, Jason Snibbe

**Affiliations:** 1. Hamilton Orthopaedics and Sports Medicine, 85 College Street, Hamilton, NY 13346, USA; 2. Department of Orthopaedics, University of Rochester Medical Center, 601 Elmwood Ave, Box 665, Rochester, NY 14642, USA; 3. Kerlan Jobe Orthopaedic Clinic, 6801 Park Terrace, Los Angeles, CA 90045, USA; 4. Snibbe Orthopaedics, Snibbe Hip Institute, 120 South Spalding Drive, Suite 40, Beverly Hills, CA 90212, USA

## Abstract

To compare the clinical outcome of patients treated with and without platelet-rich plasma (PRP) injection while undergoing arthroscopic labral repair and femoral neck osteoplasty for femoral acetabular impingement. Patients were randomized at the time of surgery to receive either an intra-articular injection of 5 cc of PRP, or an equal volume of 0.9% normal saline. All patients underwent arthroscopic labral repair and osteoplasty of the femoral neck and, at the conclusion of the case, received the injection. One week following surgery, thigh circumference (measured 10 cm distal to the tip of the greater trochanter) and the presence of ecchymosis of the thigh were recorded. Clinical outcome scores, including Non-Arthritic Hip Score, Modified Harris Hip Score and Hip Outcome Score were collected prior to surgery at 1, 3, 6 and a minimum of 12 months post-operatively. Thirty-five patients were enrolled into this study. Twenty patients received a PRP injection and 15 received a saline injection. Thigh circumference was compared pre-operatively and 1 week post-operatively. There was no significant difference between the two groups. Ecchymosis was compared between the two groups at 1 week post-operatively. Four of the 20 patients in the PRP group and 10 of the 15 in the placebo group demonstrated bruising on the lateral thigh. This was compared with a Chi-Square test and found to be statistically significant (*P* = 0.005). There was no significant difference in any of the outcome scores between the two groups. An intra-articular injection of PRP after labral repair did not improve the clinical outcome up to 1 year post-operatively in patients undergoing arthroscopic labral repair and osteoplasty of the femoral neck. Level of evidence is level I study.

## INTRODUCTION

Femoroacetabular impingement (FAI) is a recently proposed pathomechanical process that has been implicated in the development of hip osteoarthritis (OA) [1–3]. Mid-range and long-term studies on arthroscopic treatment of FAI have shown consistent, sustained benefit over time [4–6]. In athletes, arthroscopic treatment of FAI has resulted in a high rate of return to sports participation [7]. Although many treating FAI have reported positive results following surgery, the opportunity for improvement still remains. Biological agents have been used to augment soft tissue repair and bone healing for a variety of body areas [8–13].

Platelet enriched plasma (PRP) is gaining widespread popularity within the orthopedic community because of its multimodal effect on the healing properties of musculoskeletal tissues [10, 14]. PRP applications have demonstrated efficacy in the treatment of a great diversity of orthopedic conditions [15]. A systematic review by Vavken *et al*. concluded that PRP application at the time of anterior cruciate ligament (ACL) reconstruction improved outcome. However, the authors noted a large variability between studies [16]. Mei-Dan *et al*. [17] demonstrated that patients who underwent PRP injection for talus osteochondral lesions had significantly better outcomes than a cohort of patients who underwent hyaluronic acid (HA) injection. Knee OA was also shown to respond favorably to PRP injection when compared with both saline and HA in two different studies [18, 19]. Similarly, Sanchez *et al*. [20] showed that intra-articular PRP injections were effective for hip OA and reported improvements in pain and functional outcome scores at 6 months post-injection. In addition to improving the overall healing environment, PRP has been shown to display antibacterial and analgesic properties, as well as promote coagulation and hemostasis [21]. As PRP is derived from autologous blood, associated morbidity and risk is low, making this an attractive treatment adjunct for patients.

Despite evidence that PRP may positively impact healing and patient reported outcomes (PROs) for a variety of orthopedics conditions, more equivocal results have also been reported. Chahal *et al.* conducted a systematic review of the literature evaluating the use PRP as adjuvant intra-operative therapy for rotator cuff repair. The authors found no significant effect on re-tear rates or outcome scores [22]. Similarly, a recent study showed that intra-operative PRP administration after arthroscopic acetabular labral repair had no significant effect on PROs [23].

The primary aim of this study was to evaluate the influence of PRP on clinical outcomes in patients with FAI following arthroscopic labral repair and femoral neck osteoplasty. We hypothesized that patients treated with PRP would display less post-operative ecchymosis and swelling, and would show greater improvement ‘earlier’ in their post-operative course than a control population.

## MATERIALS AND METHODS

Prior to patient enrollment, IRB approval was granted. Patients were recruited from the private clinic of the senior author, who performed each procedure. All patients undergoing primary or revision arthroscopic labral repair with femoral neck osteoplasty for clinically and radiographically confirmed FAI were included in this study. Patients unwilling to participate or unable to consent to participation, as well as patients with hyperlaxity or dysplasia were excluded. Patients who underwent hip arthroscopy for an indication other than treatment of FAI were excluded from the study as well as patients with <2 mm joint space on either the anteroposterior (AP) or lateral radiographs, underlying coagulopathy or those using perioperative anticoagulants. Patients were instructed to refrain from the use of non-steroidal anti-inflammatories 3 days prior to surgery. All patients signed an approved written consent form prior to participation.

Eligible patients underwent a standardized pre-operative assessment including a detailed history and physical examination, as well as AP and cross-table lateral X-rays, and an MR-arthrogram of the affected hip. FAI subtypes (Cam, Pincer or Mixed) were designated based on radiographic parameters. Hips with retroversion or coxa profunda were described as pincer-type impingement. Acetabular retroversion was characterized by the presence of a cross-over sign on a well centered AP pelvic radiograph, where the radio-opacity of the anterior aspect of the acetabular rim crossed that of the posterior aspect of the rim before reaching the lateral edge of the sourcil. Coxa profunda was determined to be present when the floor of the fossa acetabula was at or medial to the ilioischial line [24]. Cam impingement was defined as an alpha-angle >50°. Patients with mixed impingement exhibited an alpha-angle >50 with concomitant acetabular retroversion or coxa profunda. Physical examination included range of motion and specific provocative tests (flexion abduction external rotation and flexion adduction internal rotation). Several outcome measures were obtained, including a Non-Arthritic Hip Score (NAHS), a Modified Harris Hip Score (MHHS) and Hip Outcome Score (HOS; activities of daily living (ADL) and sport subset). A random number generator was used to stratify patients into respective treatment group.

All patients underwent hip arthroscopy with ‘looped’ labral repair and femoral head–neck osteochondroplasty. The labrum was repaired with a minimum of two 2.9 mm PushLock anchors (Arthrex, Naples, FL, USA). In patients with mixed-type impingement, the overhanging acetabular rim was resected with a high-speed burr prior to labral repair. On completion of the operative portion of the case, inflow was stopped and the joint was evacuated of any residual fluid. A sealed envelope containing instructions on group assignment was then opened. Based on group assignment, patients then underwent an intra-articular injection of ∼5 cc of PRP prepared with the Accelerate Concentrating System (Exactech Biologics, Gainesville, FL, USA) or an equal volume of 0.9% normal saline. All patients had blood drawn pre-operatively to minimize potential bias.

Post-operatively, patients were permitted to bear weight as tolerated. Physiotherapy was instituted after the first post-operative visit and progressed in sequential fashion, first emphasizing motion, then strength and finally endurance and functional reconditioning. One week following surgery, patients underwent routine follow-up and at that time, thigh circumference was measured (10 cm distal to the tip of the greater trochanter) and the presence of ecchymosis of the thigh was recorded. Clinical outcome scores were collected prior to surgery and at 1 month, 3 months, 6 months and a minimum of 12 months post-operatively.

To determine an adequate sample size for the study, an *a priori* power analysis was conducted based on pilot data generated from the first 10 patients (5 PRP, 5 control). The ADL portion of the HOS at 1 month post-op was used as the primary PRO of interest [25]. Pilot data demonstrated that the standard deviation of the post-minus pre-change in ADL HOS was SD = 10 units. Based on this information, a sample size of 15 patients per group (30 patients total) would sufficiently confirm mean differences in the change from pre-op baseline of 11.0 ADL HOS units or more with 80% power, using the usual two-sided *P* < 0.05 significance criterion.

## RESULTS

Thirty-five consecutive patients were prospectively enrolled into this study. Twenty patients received a PRP injection and 15 received a saline injection at the conclusion of their surgery. The demographics of each group and FAI subtype are detailed in Table I. All patients underwent osteoplasty of the femoral neck to improve head–neck offset and, when necessary, underwent acetabular rim trimming to eliminate any acetabular-sided contribution to their structural conflict. Patients without significant acetabular rim prominence underwent minimal rim trimming to decorticate bone and promote a favorable biologic healing response. All patients in both groups underwent acetabular labral repair with a minimum of two 2.9 mm PushLocks (Arthrex). In the PRP group, 13 patients underwent chondroplasty of the acetabulum, 3 patients underwent iliopsoas release, 2 patients underwent micro-fracture and 2 patients underwent a bursectomy of the peri-trochanteric space. In the control group, 10 patients underwent chondroplasty of the acetabulum, 3 patients underwent iliopsoas release and 1 patient underwent a bursectomy of the peri-trochanteric space.

**Table I.** Demographics of the PRP and placebo groups

	PRP	Placebo
Number	20	15
Mean age (range)	34.4 (18–63)	34.9 (18–54)
Cam deformity	12	5
Mixed deformity	8	10

All patients underwent pre-operative measurement of thigh circumference. A mean value of 52.4 cm (SD = 11.2) was noted for the PRP group and 49.9 cm (SD = 6.1) in the placebo group. Of the 20 patients in the PRP group, 18 patients underwent post-operative measurement of their thigh circumference with an average of 50.1 cm (SD = 5.8). Thirteen of the 15 patients in the placebo group underwent post-operative measurement of their thigh circumference with an average of 50.1 cm (SD = 6.4). There was no significant difference between the two groups pre-operatively or post-operatively with a *P*-value of 0.445 and 0.988, respectively. In the PRP group, the thigh was an average of 0.14 cm smaller post-operatively and in the control group, the thigh was an average of 0.08 cm smaller (*P* = 0.82). At the first post-operative visit, 4 of the 20 patients in the PRP group exhibited bruising on the lateral thigh compared with 10 of 15 in the placebo group. This difference was statistically significant (*P* = 0.005).

In the PRP group, all 20 patients completed the NAHS, MHHS and HOS pre-operatively and at 1 month. At 3 months, 15 patients completed the NAHS and HOS and 14 completed the MHHS. At 6 months, 11 patients in the PRP group completed the NAHS, MHHS and HOS and at minimum of 12 months, 10 patients completed the NAHS, MHHS and HOS. In the placebo group, 15 patients completed the NAHS, MHHS and HOS at 1 month, 14 patients at 3 months, 12 patients at 6 months and 7 patients at a minimum of 12 months. Twelve patients in the PRP group had a minimum of 6 months follow-up with mean follow-up of 18.5 months and 12 patients in the placebo group had a minimum of 6 months follow-up with mean follow-up of 23.3 months. The mean values for the outcome scores are shown in Tables II–IV. There was no significant difference between the two groups at any time point.

**Table II.** Mean and SD values for ADL and sports subscale of the HOS pre-operatively and at 1 month, 3 months, 6 months and 12 months post-operatively for the PRP and placebo groups

	PRP ADL	Placebo ADL	PRP sports	Placebo sports
Pre-op	59.1 (16.6)	55.7 (22.9)	35.1 (24.2)	29.2 (25.1)
1 month	68.4 (22.0)	58.9 (24.2)	31.6 (21.0)	20.2 (20.7)
3 months	79.0 (16.4)	78.9 (19.5)	55.4 (27.1)	47.8 (29.2)
6 months	79.6 (22.8)	88.3 (7.9)	61.7 (26.8)	75.6 (27.8)
12 months	84.1 (21.8)	85.0 (25.4)	65.4 (35.4)	75.2 (39.3)

**Table III.** Mean and SD values for MHHS pre-operatively and at 1 month, 3 months, 6 months and 12 months post-operatively for the PRP and placebo groups

	PRP	Placebo
Pre-op	51.9 (14.3)	50.3 (21.1)
1 month	66.6 (21.3)	59.6 (24.7)
3 months	77.0 (18.3)	75.0 (18.4)
6 months	78.4 (22.2)	83.4 (15.5)
12 months	75.9 (21.6)	81.3 (29.6)

**Table IV.** Mean and SD values for NAHS pre-operatively and at 1 month, 3 months, 6 months and 12 months post-operatively for the PRP and placebo groups

	PRP	Placebo
Pre-op	54.9 (16.7)	52.6 (20.8)
1 month	66.3 (16.5)	59.1 (22.7)
3 months	74.1 (15.6)	77.1 (16.0)
6 months	81.3 (16.1)	87.6 (10.1)
12 months	82.0 (17.2)	80.9 (26.7)

## DISCUSSION

PRP has been used to augment or treat soft tissue injuries, tendinopathies and degenerative joint disease with varying success [16–20]. The exact mechanism of PRP is unknown, however, a multimodal effect profile is speculated. The beneficial effects of PRP applications are thought to result from anti-inflammatory properties as well as promotion of an augmented or accelerated healing response through the release of cytokines and growth factors [11].

The theoretical benefits of PRP in the setting of hip arthroscopy include biologically enhanced labral healing and an overall decrease in post-operative inflammation. Determining whether there is a measurable influence on labral healing would likely require longer follow-up and additional diagnostic imaging [magnetic resonance imaging (MRI)], which was not obtained in this study. However, quantifiable markers of post-operative inflammation were studied. Bruising and swelling are two common post-operative complaints that may adversely affect patient satisfaction in the first few days after surgery. Through its pro-thrombotic effects, PRP has the potential to limit post-operative bleeding and hematoma expansion. In this study, thigh circumference was used as a proxy for soft tissue swelling, mediated by pro-inflammatory chemokines and the clotting cascade. While differences in thigh circumferences were not detected, there was a statistically significant reduction in post-operative bruising in the PRP group. There were no significant differences in HOS, MHSS or the NAHS at any time point between study groups.

There may be several reasons why differences in clinical outcomes were not observed between groups. The PRP was delivered through a spinal needle after all arthroscopic fluid had been evacuated from the joint to prevent any significant dilution of the PRP. However, there was no way to assure the PRP delivered into the joint was bathing the repaired labrum. Furthermore, the capsule was not closed for any patients, therefore, some of the PRP may have extravasated into the surrounding soft tissues and very little actually delivered to the labrum. Egress of platelets and pro-thrombotic factors into the surrounding tissues may explain the reduction in bruising observed for patients who received PRP injections. Recent studies have shown improved outcomes and healing rates after rotator cuff repair with PRP, applied with a gel delivery medium [26]. Therefore, a more focal delivery vehicle, targeting the acetabular labrum, may further enhance functional outcomes, and could potentially represent an area of future study. Although post-operative MRIs were not obtained to investigate the structurally integrity of the labral repair site, PROs were felt to represent an appropriate proxy for successful incorporation of a repair construct.

The adjunctive use of PRP in total joint arthroplasty has been shown to decrease the post-operative transfusion requirements, presumably by accelerating hemostasis [27]. In our study we found that under the age of 50, no patient who received PRP exhibited post-operative ecchymosis. However, in control group patients under the age of 50 years old, three experienced some degree of ecchymosis. There is some evidence that efficacy of PRP may deteriorate as patients age, with concomitant down-regulation of growth factors and decreased sensitivity of receptors [28]. It would seem reasonable that factors involved in promotion of hemostasis may also experience a similar down-regulation. Alternatively, this finding may simply represent increased capillary fragility associated with age, rather than lack of efficacy from PRP.

## LIMITATIONS

One limitation of our study is the lack of complete follow-up. Only 24 patients had complete data at a minimum 6 months follow-up. This attrition led to reduced power to detect a statistically significant difference in the HOS ADL score after the 3 months follow-up point. Other limitations include a small sample size and lack of post-operative imaging to assess labral healing between the two groups. Our time points failed to uncover any difference in clinical outcomes between groups. There may have been differences in early time points, perhaps 1 week, which would not have been detected by our study design. There were also more patients in the PRP group found to have chondral injuries at the time of arthroscopy. The degree of chondral injury has been shown to impart a strong influence on clinical outcomes following arthroscopic hip surgery [4]. The grade of chondral injury was not available upon review of the operative reports to further analyze this variable. Strengths of this study include a prospective design, randomization, patient blinding and placebo control arm.

## CONCLUSION

In conclusion, the application of PRP after arthroscopic femoroplasty and labral repair did not lead to improved patient-reported outcomes, but may influence post-operative bleeding.
